# A dual cohesin–dockerin complex binding mode in *Bacteroides cellulosolvens* contributes to the size and complexity of its cellulosome

**DOI:** 10.1016/j.jbc.2021.100552

**Published:** 2021-03-18

**Authors:** Marlene Duarte, Aldino Viegas, Victor D. Alves, José A.M. Prates, Luís M.A. Ferreira, Shabir Najmudin, Eurico J. Cabrita, Ana Luísa Carvalho, Carlos M.G.A. Fontes, Pedro Bule

**Affiliations:** 1 Faculty of Veterinary Medicine, CIISA – Centre for Interdisciplinary Research in Animal Health, University of Lisbon, Pólo Universitário do Alto da Ajuda, Avenida da Universidade Técnica, Lisboa, Portugal; 2 UCIBIO, Departamento de Química, Faculdade de Ciências e Tecnologia, Universidade Nova de Lisboa, Caparica, Portugal; 3 Randall Centre for Cell and Molecular Biophysics, King’s College London, London, United Kingdom; 4 Research and Development, NZYTech Genes & Enzymes, Lisboa, Portugal

**Keywords:** cellulosome, cohesin, dockerin, dual-binding, protein complex, crystal structure, cellulose, cellulase, *A. cellulolyticus*, Acetivibrio (Hungateiclostridium) cellulolyticus, *B. cellulosolvens*, *Bacteroides* (Pseudobacteroides) cellulosolvens, *C. cellulolyticum*, *Clostridium* (Hungateiclostridium) cellulolyticum, *C. thermocellum*, *Clostridium* (Hungateiclostridium) thermocellum, CAZymes, Carbohydrate Active enZymes, Coh, Cohesin, Doc, Dockerin, IMAC, Immobilized Metal-ion Affinity Chromatography, ITC, Isothermal Titration Calorimetry, NGE, Nondenaturing Gel Electrophoresis, *R. flavefaciens*, *Ruminococcus flavefaciens*, rmsd, root-mean-square deviation, SLH, S-layer homology

## Abstract

The Cellulosome is an intricate macromolecular protein complex that centralizes the cellulolytic efforts of many anaerobic microorganisms through the promotion of enzyme synergy and protein stability. The assembly of numerous carbohydrate processing enzymes into a macromolecular multiprotein structure results from the interaction of enzyme-borne dockerin modules with repeated cohesin modules present in noncatalytic scaffold proteins, termed scaffoldins. Cohesin–dockerin (Coh-Doc) modules are typically classified into different types, depending on structural conformation and cellulosome role. Thus, type I Coh-Doc complexes are usually responsible for enzyme integration into the cellulosome, while type II Coh-Doc complexes tether the cellulosome to the bacterial wall. In contrast to other known cellulosomes, cohesin types from *Bacteroides cellulosolvens*, a cellulosome-producing bacterium capable of utilizing cellulose and cellobiose as carbon sources, are reversed for all scaffoldins, *i.e.*, the type II cohesins are located on the enzyme-integrating primary scaffoldin, whereas the type I cohesins are located on the anchoring scaffoldins. It has been previously shown that type I *B. cellulosolvens* interactions possess a dual-binding mode that adds flexibility to scaffoldin assembly. Herein, we report the structural mechanism of enzyme recruitment into *B. cellulosolvens* cellulosome and the identification of the molecular determinants of its type II cohesin–dockerin interactions. The results indicate that, unlike other type II complexes, these possess a dual-binding mode of interaction, akin to type I complexes. Therefore, the plasticity of dual-binding mode interactions seems to play a pivotal role in the assembly of *B. cellulosolvens* cellulosome, which is consistent with its unmatched complexity and size.

Recycling of photosynthetically fixed carbon is a crucial microbial process, critical to the cycling of carbon between plants, herbivores, and microbes. *Bacteroides (Pseudobacteroides) cellulosolvens* is a mesophilic, anaerobic bacterium capable of degrading crystalline cellulose (1, 2). Similar to other bacteria such as *Clostridium (Hungateiclostridium) thermocellum* and *Acetivibrio (Hungateiclostridium) cellulolyticus*, *Bacteroides cellulosolvens* produces an extracellular multimodular cellulolytic complex—the cellulosome—responsible for the degradation of the plant cell wall (3). Noteworthy, *B. cellulosolvens* has the most intricate cellulosome so far described, conceivably capable of congregating up to 110 carbohydrate-active enzymes in a cell-associated mega-Dalton complex (Fig. 1) (4).

**Figure 1:**
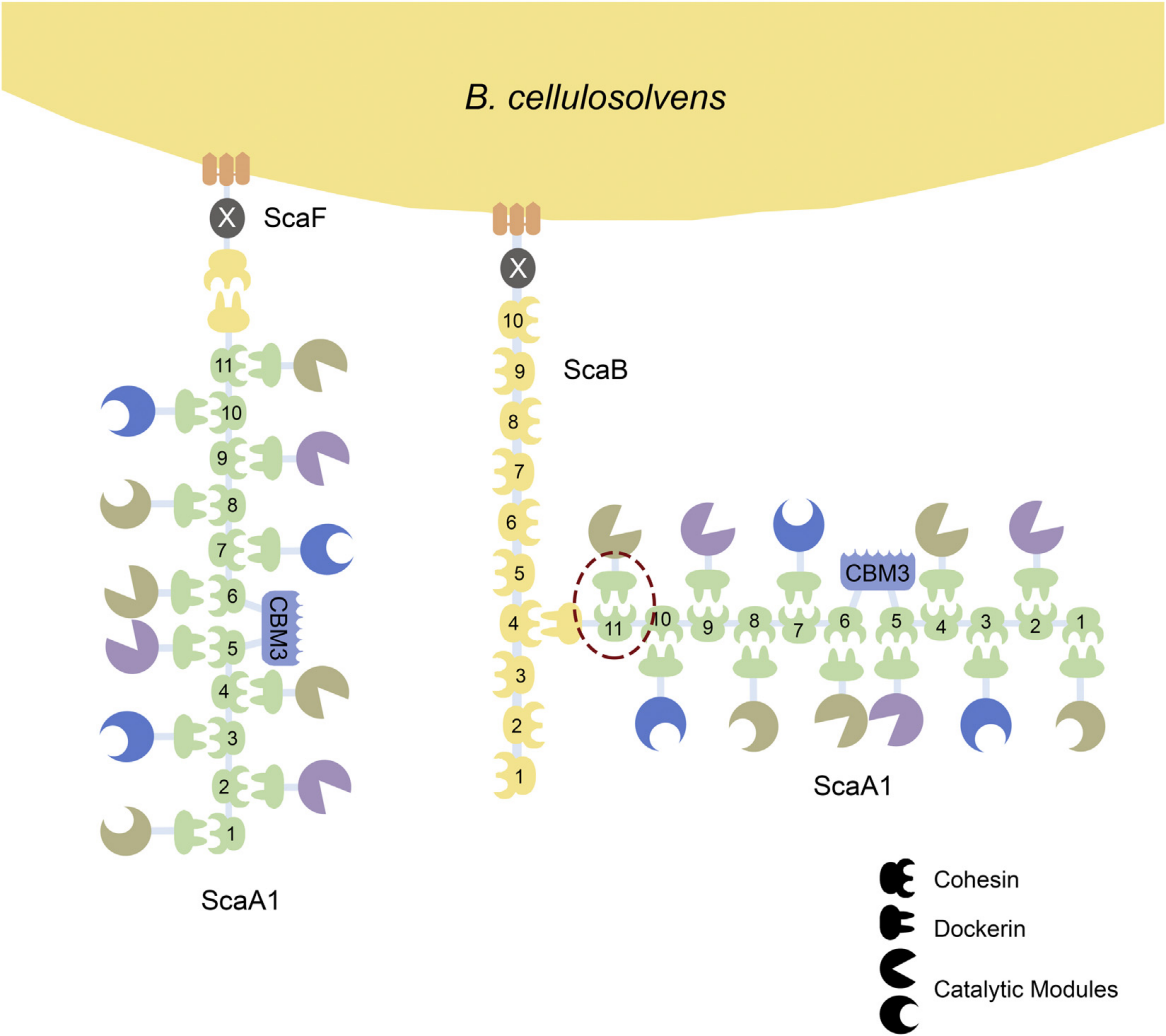
**Schematic representation of two possible *B. cellulosolvens* cellulosomal architectures.** The cohesins and dockerins are color-coded to highlight the different Coh-Doc specificities. Doc-containing enzymes are incorporated into the ScaA1 scaffoldin through interaction with the 11 ScaA1 Cohs (*light green*). ScaA1 can then bind to anchoring scaffoldins ScaF or ScaB, tethering the cellulosome to the wall (*yellow interactions*). ScaF can only interact with a single ScaA1 scaffoldin, originating a cellulosomal unit with 11 enzymes. On the other hand, ScaB can integrate up to ten different ScaA1 units, generating a cellulosome with 110 enzymes. ScaA1 possesses an internal family 3 CBM unit (*light blue*) for substrate targeting. ScaF and ScaB both possess a SLH module (*orange*) for cell surface attachment, with an adjacent X-module (*gray*) with unknown function. The interaction here described (*Bc*CohScaA1_11_-DocCel48) is highlighted with a *dashed red circle*.

Cellulosomes are built around a primary noncatalytic protein scaffold, named scaffoldin, bearing reiterated cohesin (Coh) modules that serve as protein–protein interaction targets to dockerin (Doc) modules found in an extensive repertoire of independent Carbohydrate-Active enZymes (CAZymes). The ability to gather a large number of diverse enzymes into the cellulosome presumably provides these anaerobic microorganisms an important advantage in their competitive ecological niches, through complementary and synergic enzyme cooperation, while also promoting enzyme stability (5). The cellulosome’s Coh-Doc protein:protein interactions constitute the primary driving force for cellulosomal assembly and are among nature’s strongest protein:protein interactions (Ka > 10^9^ M^−1^). Also, due to a distinctive twofold internal symmetry, Doc modules can potentially bind their cognate Cohs in two different orientations, by rotating 180° with respect to its protein ligand (6). This is known as a dual-binding mode, as opposed to a single-binding mode that occurs when only one of the Doc interfaces supports the formation of the Coh-Doc complex (7). A sequence-based classification of Cohs and Docs distinguishes type I and type II interactions as major categories (8). In the archetypal cellulosome of *Clostridium thermocellum*, the assembly of the different enzymes into the main scaffoldins is mediated by type I Coh-Doc interactions with a dual-binding mode, whereas the anchoring of the scaffoldin to the bacterial cell wall is achieved through type II single-binding mode Coh-Doc interactions (5, 9). Although there are noteworthy exceptions, such as a generalized Coh-Doc dual-binding mode in the cellulosome of *Acetivibrio cellulolyticus* (8, 10) or, conversely, a ubiquitous single-binding mode found in *Ruminococcus flavefaciens* (11), this has been considered the rule.

Based on the almost complete genome sequence of *B. cellulosolvens* (12), Zhivin *et al.* (4) performed a seminal study on the architecture and functional organization of the most complex cellulosome described so far. Their work has identified 31 different scaffoldins, many of which lack any known cell-surface binding domains, thus supporting an extensive putative cell-free cellulosome system. Besides a novel classification of Coh-Doc pairings, named type R, another striking feature of this cellulosome is a reversal of Coh types found in scaffoldins. Unlike in other species, *B. cellulosolvens’*primary scaffoldin recruitment of Doc-bearing enzymes is mediated by type II Coh-Doc interactions, while anchoring scaffoldins rely on type I Coh-Doc complexes for cell wall attachment. The primary scaffoldin subunit of *B. cellulosolvens*, termed ScaA1, like other primary scaffoldin proteins, incorporates a carbohydrate-binding module (family 3 CBM), but is unusually composed of 11 type II Cohs and a C-terminal type I Doc that does not have an associated X module, which is a known type II Coh-Doc interaction stabilizer (3). Cell-surface anchoring of ScaA1 occurs *via* interaction of its single type I Doc with one of the ten type I Cohs found in the ScaB anchoring scaffoldin. Anchoring to the peptidoglycan-associated polymers from the bacterium cell surface likely results from a noncovalent interaction with ScaB’s S-layer homology (SLH) domain (13). This SLH-mediated interaction is likely aided by an adjacent X module, another *B. cellulosolvens* peculiarity (4). While the functional implications of the reversed specificity of Coh types in *B. cellulosolvens* cellulosome architecture remain unclear, the actual structural determinants of specificity of its Coh-Doc type II interactions are also currently unknown. This knowledge gap is relevant considering that, although known cellulosome Coh-Doc structures share a remarkable overall conservation of structural topology, the major determinants for interspecies and intraspecies barriers, as well as for the binding mode, depend on very subtle amino acid residue differences (14).

The structure of *B. cellulosolvens* isolated 11th type II Coh module of *Bc*ScaA1 (PDB code: 1tyj (15)), *Bc*ScaA1-CBM3 (PDB code: 2xbt, (16)), and seventh type I Coh module of *Bc*ScaB (PDB code: 4ums, (10)) were previously reported. The latter report suggested a dual-binding mode for *B. cellulosolvens* cellulosomal cell anchoring. This presumed plasticity for cell anchoring binding mode is similar to that found on *A. cellulolyticus* Coh-Doc interactions involving the primary scaffoldin (*Ac*ScaA), a unique adaptor scaffoldin (*Ac*ScaB) and several anchoring scaffoldins (*Ac*ScaC, *Ac*ScaD, and *Ac*ScaF) (PDB codes 4u3s/4wi0 (8), and 4uyp/4uyq, (10). All of these are in contradiction to the canonical single-binding mode found for cellulosome cell anchoring in *C. thermocellum* (PDB code 2b59, (17). The 11th type II Coh structure of *Bc*ScaA1 shows an overall fold similarity with several type II Cohs from *A. cellulolyticus*, namely with *Ac*CohScaB3 (PDB code 4u3s), with a Q-score of 0.79 and root-mean-square deviation (rmsd) of 1.22 Å over 163 aligned Cα residues. It also bears close homology to the type II *Ct*CohScaF (PDB code 2b59) with a Q-score of 0.77 and rmsd of 1.40 Å against 164 aligned Cα residues. They all share the characteristic α-helical crowning between strands 6 and 7 and the two singular β-flaps that disrupt strands 4 and, particularly, strand 8 where its 12 residues are flanking the type II Coh Doc-binding plateau.

The elucidation of *B. cellulosolvens* cellulosome assembly and the reversal of Coh types found in its scaffoldins hinge upon the availability of type II CohScaA-Doc complex structures to understand the consequences of this unusual arrangement. Here is reported the crystal structure of the type II 11th Coh of the primary scaffoldin of *B. cellulosolvens* in complex with the Doc module of a glycoside hydrolase of family 48, *Bc*CohScaA1_11_-DocCel48 (PDB code: 2y3n). A detailed binding characterization informed by the structural data has also been carried out, which allowed the identification of the molecular determinants of Coh-Doc interaction and suggested a typical dual-binding mode for cellulosome enzyme assembly, thus agreeing with the common paradigm for a flexible arrangement, as found on *C. thermocellum* (6). In light of this report and considering the putative dual-binding mode for scaffoldin assembly in both *B. cellulosolvens* and *A. cellulolyticus* (10), it is fair to conclude that the single *versus* dual-binding mode in Coh-Doc complexes is independent of type classification. It also does not seem to be a matter of enzyme assembly *versus* cell-anchoring/scaffoldin assembly. Rather, it might be related to the size and complexity that is possible to achieve in a single unit within a cellulosomal system, with larger cellulosomes requiring a higher degree of flexibility for proper assembly and access to substrate.

## Results and discussion

A critical factor to understand the mechanism of cellulosome assembly of *B. cellulosolvens* is the availability of an X-ray crystal structure of the type II Coh-Doc interaction, central to CAZyme assembly around the primary *Bc*ScaA1 scaffoldin. *Escherichia coli* coexpression strategies for the production and purification of Coh-Doc complexes were thus used to obtain good-quality crystals of highly pure protein complexes of the type II 11th Coh of the primary scaffoldin of *B. cellulosolvens* (*Bc*CohScaA1_11_), in complex with the Doc module of a glycoside hydrolase of family 48 (DocCel48). A previous study described the structure of this Coh in its unbound form (PDB code: 1tyj (15)), and solving its structure in complex with a bound Doc would allow probing structural differences arising from Coh binding to a Doc partner. The chosen Doc module belongs to one of the most abundant cellulosomal CAZymes, GH48 cellobiohydrolase, and was previously reported to bind Cohs from ScaA1 (4, 18).

### Expression and crystallization of *B. cellulosolvens* Coh-Doc complex

The high degree of sequence conservation between the DocCel48’s two dockerin repeats suggests the existence of two cohesin-binding interfaces, thus supporting a dual-binding mode. This implies that two different complex conformations could be present in solution, which would likely compromise protein crystallization due to a lack of unit cell homogeneity. It is well established that residues at relative positions 10 and 11 of each of the two Doc duplicated segments play a key role in Coh recognition and act as specificity determinants (residues #17, #18, #50, and #51 of the construct used in this work) (9). Thus, a DocCel48 mutant was designed to force binding through a single interface, promoting homogeneity of the purified protein. The mutations used for the crystallization experiments were designed to replace the putative recognition residues in relative positions 10 and 11 of the C-terminal Doc repeat (Met50 and Ala51) with those of the *B. cellulosolvens* ScaA type I Doc (Ser-Asp), rather than the commonly applied alanine substitution. These amino acid changes were chosen based on the lack of cross-reaction between type I and type II Coh-Doc complexes. The sequence of the resulting Doc is displayed in Table S2. This strategy allowed us to obtain large yields of highly pure Coh-Doc complexes for crystallization, which led to the production of well-diffracting crystals.

### Structure of a type II Coh-Doc complex from *B. cellulosolvens*

A molecular replacement strategy was used to solve the complex’s structure, using the available *Bc*CohScaA1_11_ structure as an input model (PDB code: 1tyj (15)). This yielded a solution with two Cohs in the asymmetric unit. Successive rounds of automated ARP/wARP (19) and manual COOT (20) adjustments to build the Doc modules in both crystallographic Coh-Doc dimer complexes resulted in a final REFMAC5 (21) refined structure at 1.90 Å resolution. The two molecules of the *Bc*CohScaA1_11_-DocCel48 heterodimer share 299 water molecules and each Doc is coordinating two calcium (Ca^2+^) ions. The Coh-Doc complex includes residues 2073 to 2242 from *Bc*CohScaA1_11_ (Nonredundant RefSeq accession number AAG01230), 683 to 752 from *Bc*DocCel48A (Nonredundant RefSeq accession number WP_050753099). The structure belongs to the monoclinic space group *P*12_1_1 with unit cell dimensions of *a* = 43.4 Å, *b* = 116.1 Å, *c* = 45.2 Å, and β = 112.5°. Due to disorder, some of the dockerin’s helix 2 residues could not be modeled, namely those between Gly32 and Asn37 of chain B and 15 residues from Ala30 to Asn44 in chain D. Some C- and N-terminal residues, including the 6-histidine tag, are also absent in all four chains. The final model was deposited in the Protein Data Bank under accession code 2y3n. Data collection and refinement statistics are shown in Table 1.

**Table 1:** X-ray crystallography data collection and refinement statistics for *Bc*CohScaA1-DocCel48

Data quality	*Bc*CohScaA1-DocCel48
Cell dimensions, Å	*a* = 43.4
	*b* = 116.1
	*c* = 45.2
	β = 112.5°
Space group	*P*12_1_1
X-ray source	ESRF, ID14-EH1
Wavelength, Å	0.934
Resolution of data (outer shell), Å	41.74–1.90 (2.00–1.90)
R*pim* (outer shell)^a^	0.073 (0.278)
Rmerge (outer shell)^a^	0.090–0.051 (0.329)
Mean I/σ (I) (outer shell)	15.0 (3.9)
Completeness (outer shell), %	83.9 (66.4)
Multiplicity (outer shell)	2.40 (2.2)
Structure quality	
N° of protein atoms (AU)	3765
N° calcium atoms	4
N° solvent waters	299
Resolution used in refinement, Å	1.90
Rwork/Rfree,%^b^	16.3/22.5
Average temperature factors, Å^2^	
Main chain (CohA, DocB, CohC, DocD)	21.3, 31.0, 21.2, 44.3
Side chain (CohA, DocB, CohC, DocD)	24.4, 33.0, 24.2, 47.7
Calcium atoms (B1, B2, D1, D2)	25.6, 22.9, 27.3, 61.84
Solvent waters	39.3
RMS deviations	
Bond lengths, Å	0.022
Bond angles, °	1.696
Ramachandran’s plot analysis	
Favorable, %	96.1
Allowed, %	3.6
Outlier, %	0.2
PDB accession code	2y3n

Values in parenthesis are for the highest resolution shell.

a ${\text{R}}_{merge}=\frac{\sum_{hkl}\sum_{i=1}^{n}\left|I_{i}\left(hkl\right)-\bar{I}\left(hkl\right)\right|}{\sum_{hkl}\sum_{i=1}^{n}I_{i}\left(hkl\right)}$, where *I* is the observed intensity, and $\bar{I}$ is the statistically-weighted average intensity of multiple observations. ${\text{R}}_{p.i.m.}=\frac{\sum_{hkl}\sqrt{1/\left(n-1\right)}\sum_{i=1}^{n}\left|I_{i}\left(hkl\right)-\bar{I}\left(hkl\right)\right|}{\sum_{hkl}\sum_{i=1}^{n}I_{i}\left(hkl\right)}$, a redundancy-independent version of R_merge_.

b ${\text{R}}_{work}=\frac{\sum_{hkl}\left|\left|F_{obs}\left(hkl\right)|-|F_{calc}\left(hkl\right)\right|\right|}{\sum_{hkl}\left|F_{obs}\left(hkl\right)\right|}$, where |F_calc_| and |F_obs_| are the calculated and observed structure factor amplitudes, respectively. (R_free_ is calculated for a randomly chosen 5% of the reflections).

#### *Bc*CohScaA1_11_ structure in the complex

**Figure 2:**
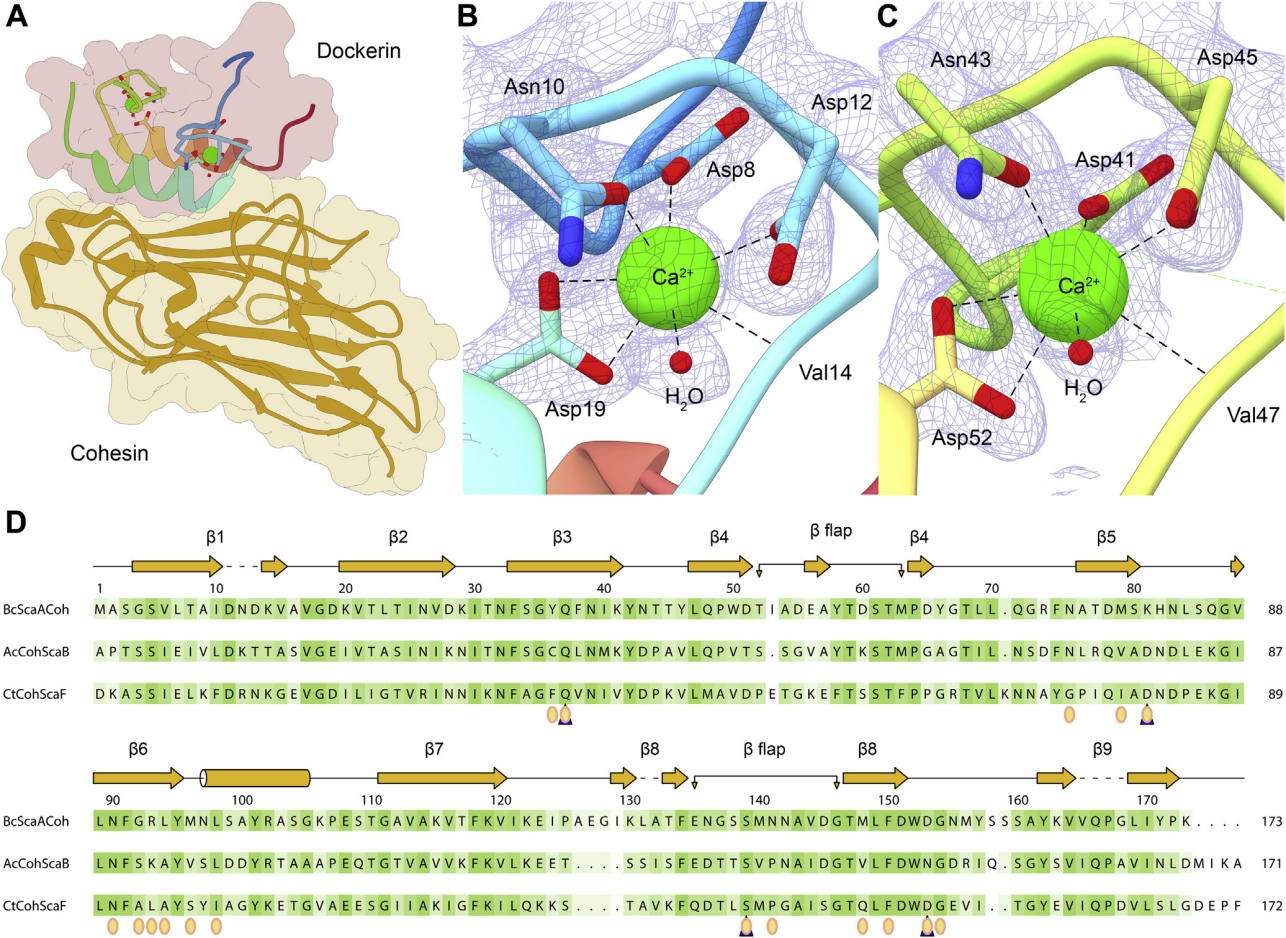
***Bc*CohScaA1**_**11**_**-DocCel48’s structure.***A*, bird’s-eye view of the *Bacteroides cellulosolvens* type II complex *Bc*CohScaA1_11_-DocCel48 in *ribbon* representation, with the dockerin color-ramped from *N*-*terminus* (*blue*) to *C*-*terminus* (*red*) and the cohesin in *gold*. The van der Waals surfaces of both modules are depicted in *transparent coloring*. *B*, detailed view of the N-terminal calcium coordinating loop. *C*, detailed view of the C-terminal calcium coordinating loop. Calcium coordinating residues are depicted in *stick* representation. Hydrogen bonds are shown as *dashed lines*, water molecules as *red spheres*, and calcium ions as *green spheres*. The coordinating residues and calcium ions are surrounded by a *mesh* representation of the Refmac5 maximum-likelihood σA-weighted 2Fo−Fc electron density map contoured at 1σ (0.46 electrons/A^3^). *D*, multiple sequence alignment of *Bc*CohScaA1_11_ with other type II cohesins from *A. cellulolyticus* (*Ac*CohScaB) and *C. thermocelum* (*Ct*CohScaF). A *cartoon* representation of *Bc*CohScaA1_11_’s secondary structure is displayed in *orange*, above the alignment. The sequences were aligned using the Clustal Omega tool and further processed with ALINE. Colouring according to similarity was implemented with ALINE (37): *dark green*, identical residues; *green* to *white*, lowering color-ramped scale of conservation. Residues involved in hydrogen bonding with *Bc*DocCel48 are marked with a *blue triangle* and those establishing hydrophobic contacts with *yellow circles*.

The cohesin domain of the type II Coh11-Doc complex of *B. cellulosolvens* shows the typical flattened, elongated 9-stranded *β*-barrel jelly-roll topology (Fig. 2*A*) with a highly hydrophobic core. Similar to the *C. thermocellum* structure, the nine *β*-strands define two *β*-sheets—the first *β*-sheet is defined by strands 8-3-6-5 (front face) and the second is defined by strands 9-1-2-7 (back face). The common *α*-helical crowning observed between strands 6 and 7 and the two *β*-flap regions that disrupt the normal progression of strands 4 and 8 are maintained (10, 11, 15), with the latter making further contact with the dockerin counterpart. Comparing this structure with that of the unbound *Bc*CohScaA1_11_ (1tyj) shows that, globally, the Coh does not undergo significant conformational changes upon binding, as revealed by the low rmsd value (0.66 Å for 166 Cα atoms) between both structures. Nonetheless, some differences can be found on the binding plateau that better accommodates the ligand partner, namely on the β-strand 8 loop (defined by residues 136 and 145) as indicated by a larger rmsd value of 1.35 Å between 11 atom pairs. The closest *Bc*CohScaA1_11_ functionally relevant structural homologues according to the PDBeFold server (http://www.ebi.ac.uk/msd-srv/ssm/) are type II cohesins from *A. cellulolyticus* ScaB (PDB accession codes 3bwz and 4u3s), responsible for the anchoring between adaptor scaffoldin ScaB and main scaffoldin ScaA through a dual-binding mode interaction. These protein modules were matched with a Z-score of 11.4, rmsd of 1.18 Å, sequence identity of 44% over 163 aligned residues and Z-score of 12.6, rmsd of 1.22 Å, sequence identity of 40% over 162 aligned residues, respectively. The type II cohesin from *C. thermocellum*’s ScaF (SdbA) also shares a high degree of structural homology with *Bc*CohScaA1_11_ (PDB 2b59), Z-score of 12.3, rmsd of 1.40 Å, sequence identity of 30% over 164 aligned residues. Thus, the dual- and single-binding Coh structures show a high degree of sequence and structural conservation (Fig. 2*D*), which is curious but not unexpected as the dual *versus* Single-binding mode is determined by the nature of the dockerin partner.

#### Type II Doc structure in the complex

The dockerin domain of the *B. cellulosolvens* type II Coh-Doc complex reveals a classic Doc structure, composed of two loop *α*-helix motifs (EF-hand like motifs) each with a bound calcium ion, separated by a 12-residue unstructured linker (7, 11, 22, 23) (Fig. 2*A*). Helix 1 is formed by residues Asn16–Ser26, and helix 3 is formed by residues Ser50–Phe60. Helices 1 and 3 are arranged in an antiparallel orientation that places the two calcium ions in opposite sides of the dockerin module, similar to that observed for other dockerins. The calcium ions are coordinated in a typical octahedral geometry in both EF-hand motif loops (Fig. 2, *B* and *C*). The first calcium ion is located near the *N*-*terminus* of the dockerin and is coordinated by residues Asp8 (Oδ1), Asn10 (Oδ1), Asp12 (Oδ1), Val14 (backbone carbonyl), Asp19 (Oδ1 and Oδ2), and a water molecule. The second calcium is coordinated by Asp41 (Oδ1), Asn43 (Oδ1), Asp45 (Oδ1), Val47 (backbone carbonyl), Asp52 (Oδ1 and Oδ2), and a water molecule. The 12-residue linker region between helices 1 and 3 (Phe27-Asn49) shows a large degree of mobility, making it difficult to define several residues. This is particularly evident in the dockerin at chain D, where the calcium ion has a B-factor of 62 Å^2^ and calcium coordinating residues Asp41 and Asn43 could not be defined. Since the type II complex of *B. cellulosolvens* lacks the X module, which is thought to help stabilize the cohesin–dockerin interaction in type II complexes, it is possible that for the correct assembly of this complex, the presence of an adjacent module is required.

**Figure 3:**
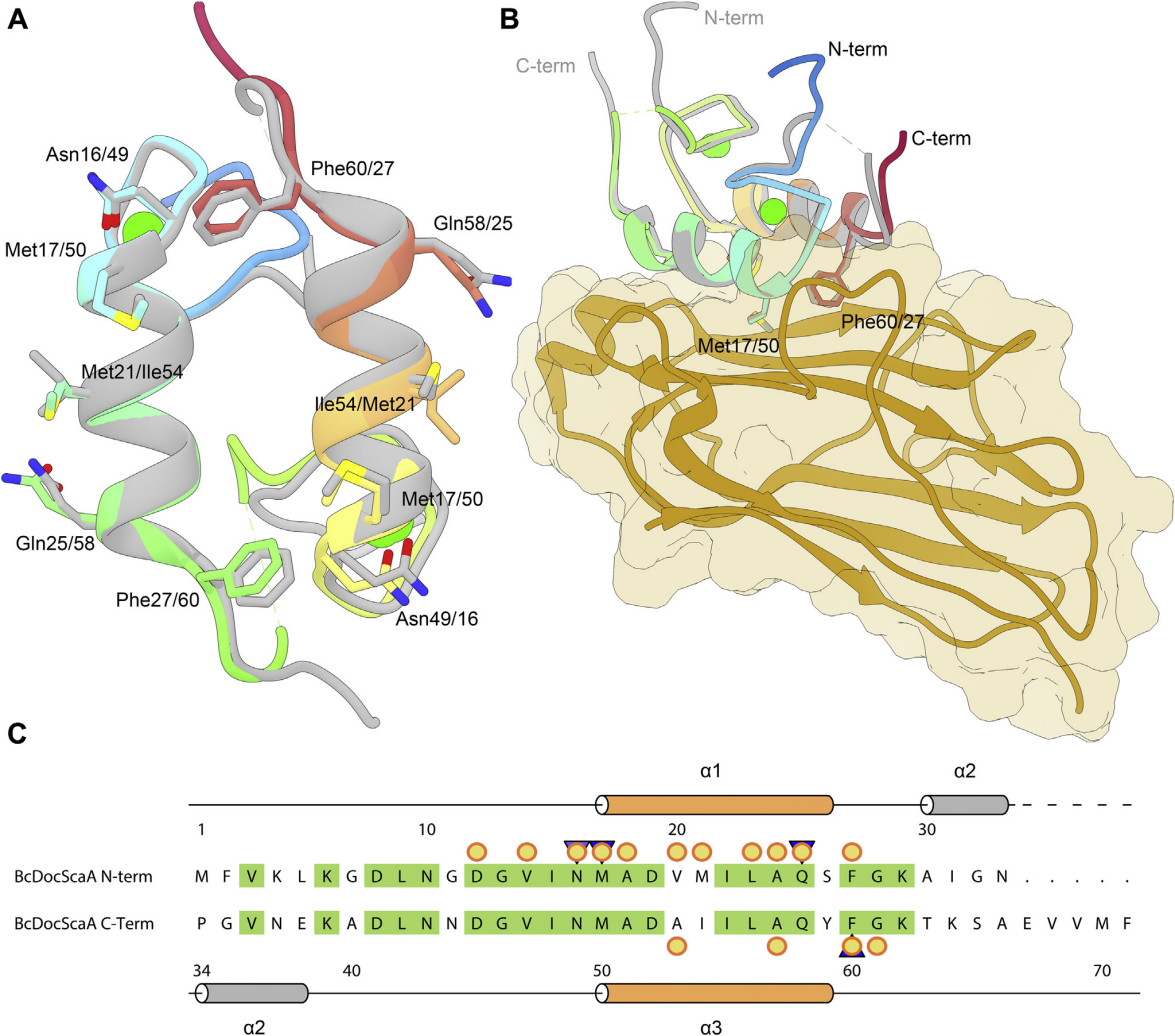
**Highly symmetric nature of *Bc*DocCel48 supports a dual-binding mode of interaction.***A*, *Bc*DocCel48’s structure overlaid with a 180° rotated version of itself (*gray*), showing conservation of key Coh interacting residues. *B*, overlaying *Bc*CohScaA1_11_-DocCel48 with a complex with a rotated dockerin (*gray*), shows that the overall structure of the complex as well as key contacts are maintained. The dockerin is shown color-ramped from *N*-*terminus* (*blue*) to *C*-*terminus* (*red*) and the cohesin in *gold*. The van der Waals surface of the cohesin is depicted in *transparent coloring*. *C*, primary structure alignment of the N-terminal half of the dockerin with its C-terminal half, showing a remarkable symmetry provided by a high degree of conservation. A *cartoon* representation of *Bc*DocCel48’s secondary structure is displayed in *orange*, above the alignment. The sequences were aligned using the Clustal Omega tool and further processed with ALINE (37). Conserved residues are colored in *green*. Residues involved in hydrogen bonding with *Bc*CohScaA1_11_ are marked with a *blue triangle* and those establishing hydrophobic contacts with *yellow circles*. The second alpha-helix could not be modeled in the structure due to disorder but is shown in *gray* in panel *C*, as it is a highly conserved feature of bacterial dockerins.

**Figure 4:**
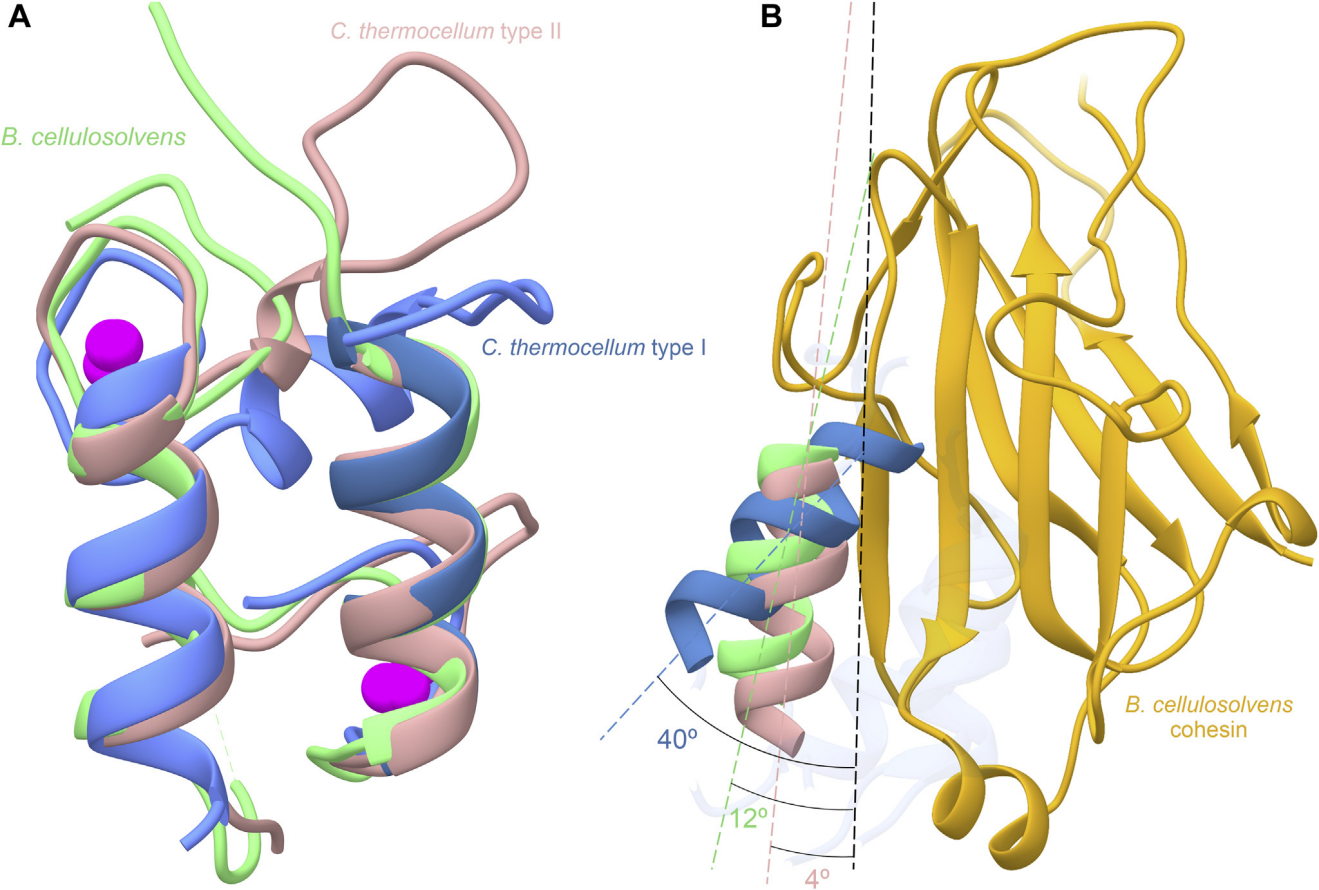
***Bc*CohScaA1**_**11**_**-DocCel48 type II interaction shares traits with both canonical type I and type II complexes.***A*, overlay of *Bc*DocCel48 (*light green*) with a type I dockerin (*blue*) and a type II dockerin (*pink*) from *C. thermocellum* showing that, despite some subtle differences, there is an overall tertiary structure conservation. *B*, position of the “non-dominant” helix (the helix which is further removed from the binding interface) of *Bc*DocCel48 (*light green*), a type I dockerin from *C. thermocellum* (*blue*), and a type II dockerin from *C. thermocellum* (*pink*), relatively to their cohesin partners. The image was obtained by superposing the cohesins of *Bc*CohScaA1_11_-DocCel48, a type I *C. thermocellum* complex (PDB: 1ohz), and a type II *C. thermocellum* complex (PDB: 5k39). The cohesin shown is *Bc*CohScaA1_11_ (in *gold*). The angle of the rotation was determined by measuring the angles formed by the helices and the longitudinal orientation of the cohesin’s β-strands at the binding interface. This reveals that the angle formed between *Bc*DocCel48 nondominant helix and the cohesin stands between those seen in the canonical type I and type II complexes of *C. thermocellum*.

The structure of *B. cellulosolvens* type II Doc presents a functionally relevant internal symmetry, as illustrated in Figure 3. This internal symmetry is reflected by the low rmsd values between both helices (0.62 Å for 24 Cα atoms). When *B. cellulosolvens* dockerin structure was compared with both Xyn10 B type I (PDB code: 1ohz) and the CipB type II (PDB code: 5k39) dockerins from *C. thermocellum*, it is clear that helices 1 and 3 are highly homologous, with low rmsd values (1.20 Å for 26 Cα atoms and 0.75 Å for 39 Cα atoms for the type I and type II modules, respectively) (Fig. 4*A*). Interestingly, the first calcium-binding region is shorter than in the CipB complex, bringing it closer to the type I structure. Moreover, the internal symmetry between helices 1 and 3 exists not only at the tertiary structure level, as in the case of the CipB type II complex, but also at the primary sequence level (Fig. 3*C*), as in the type I Xyn10B module. This is a crucial feature for the dual-binding mode mechanism.

#### The complex interface—an alternative binding mode

**Figure 5:**
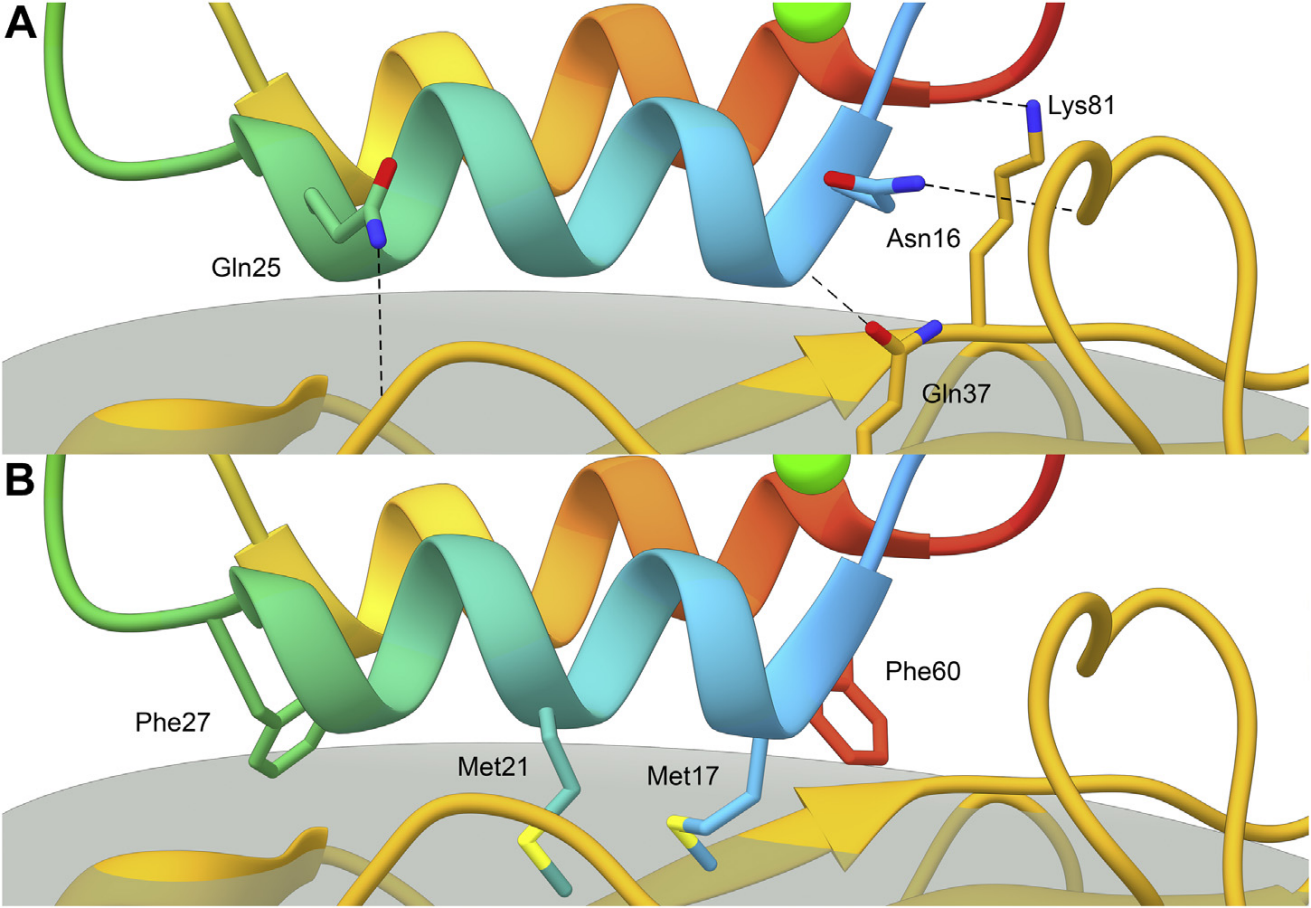
**Cohesin–dockerin interface of *Bc*CohScaA1**_**11**_**-DocCel48.** Structure of *Bc*CohScaA1_11_-DocCel48 complex with a detailed view of the Coh-Doc interface showing the main polar interactions (panel *A*) and main hydrophobic contacts (panel *B*). In both panels, the most important residues involved in Coh-Doc recognition are depicted in *stick* representation. *Dashed black lines* represent hydrogen-bond interactions. The Doc is shown color-ramped from *N*-*terminus* (*blue*) to *C*-*terminus* (*red*). The Coh is shown in *gold*. Ca^2+^ ions are depicted as *green spheres*. In both panels, the *transparent gray disk* marks the plane defined by the 8-3-6-5 β-sheet, where the β-strands form a distinctive Doc-interacting plateau.

The *Bc*CohScaA1-DocCel48 cohesin–dockerin interface comprises mainly one face of the cohesin (defined by strands 8-3-6-5) and helices 1 and 3 of the dockerin. The contacts were calculated using the PDBSum server. Among these interactions there are several hydrophobic contacts and a few hydrogen bonds (Fig. 5), which occur mainly between helix 1 of the dockerin and the cohesin (Table 2 and Table S1). This indicates a preferential helix for the formation of the complex as in the case of the type I *C. thermocellum* complex. When compared with other Coh-Doc complexes, namely with the type I (PDB code: 1ohz) and type II (PDB code: 5k39) complexes from *C. thermocellum*, the position of the dockerin relatively to the cohesin lays midway between both structures. In *C. thermocellum’s* type I complex, the dockerin helix that is further away from the binding interface is tilted by about 40° relative to the longitudinal orientation of the cohesin’s β-strands, while in its type II counterpart they run almost parallel, with a slight 4° rotation of the helix. In comparison, the same helix in *B. cellulosolvens’*type II DocCel48 is tilted by about 12° (Fig. 4*B*). Consequently, this helix forms fewer contacts with the cohesin than in the *C. thermocellum’s* type II complex but a more than in the type I. Interestingly, there are no hydrogen bond contacts between side chains of both modules. All H-bonds present in the binding interface are either water-mediated or involve a backbone atom of either the cohesin or the dockerin. Thus, the data suggest that hydrophobic interactions are highly dominant in the formation of this complex (Table 2 and Table S1).

**Table 2:** Main polar contacts between *Bc*CohScaA1 and *Bc*DocCel48 M2 mutant

Direct hydrogen bonds					
#	CohScaA_11_		Distance (Å)	Dockerin	
	Residue	Atom		Residue	Atom
1	Gln37	Oε1	2.74	Met17	N
2	Lys81	NZ	3.06	Phe60	O
3	Ser139	O	2.96	Asn16	Nδ2
4	Asp153	O	2.82	Gln25	Nε2

Water-mediated hydrogen bonds								
#	CohScaA_11_		Distance (Å)	H2O		Distance (Å)	Dockerin	
	Residue	Atom			Atom		Residue	Atom
1	Gln37	Nε2	3.09	H2O11	O	3.17	Gly61	N
2	Gln37	Nε2	3.09	H2O11	O	2.70	Ile15	O
3	Ser99	N	3.58	H2O50	O	2.80	Ala24	O
4	Asn141	Oδ1	2.73	H2O100	O	2.71	Asn16	Nδ2
5	Asn141	Oδ1	2.73	H2O100	O	3.11	Asn12	Oδ2
6	Thr77	Oγ1	2.78	H2O239	O	2.73	Ser50	Oγ

Table was made using data from the PDBSum server.

### *Bc*CohScaA1_11_-DocCel48’s binding mechanism: Dual-binding mode in a type II Coh-Doc complex

The contribution of *Bc*DocCel48’s binding surface residues for Coh recognition was initially probed through nondenaturing gel electrophoresis. Residues establishing direct hydrogen bonds as well as those with the most extensive hydrophobic contacts were mutated to alanine and the resulting variants were tested against *Bc*CohScaA1. Because of the possible dual-binding mode, all mutations were performed on both helices, at equivalent positions. The data revealed that out of all *Bc*DocCel48 derivatives, the M17A/M50A mutant and the F27A/F60A mutant had the most impact on binding. To gain further insights into the driving forces of Coh-Doc recognition, the binding thermodynamics of *Bc*DocCel48 to *Bc*CohScaA1_11_ were assessed by isothermal titration calorimetry (ITC) at 308 K (Table 3, Fig. S1). The results, presented in Table 3, revealed that indeed Met17/Met50 and Phe27/Phe60 are the most important dockerin residues for cohesin interaction, as their mutation completely abolishes binding of the two modules. The structure of the complex also shows the dockerin’s Met21 establishing a considerable number of hydrophobic interactions (Table S1) with the cohesin interface. This residue’s placement near key contacting residue Met17, in a highly hydrophobic pocket formed by Coh’s residues Leu94, Leu98, and Phe150, would suggest that Met21 also plays a significant role in the interaction between the two modules. Significantly, this residue is not conserved on helix 3, where it is replaced by an isoleucine (Ile54). Whether this contact is indeed a key component for the interaction (which would contradict the dual-binding mode hypothesis) or not was assessed using the M21A/I54A mutant variant. ITC experiments against the cohesin gave similar affinity to that of the wild-type dockerin, suggesting that these residues are not essential for the interaction. Finally, the N16A/N49A and Q25A/Q58A dockerin mutations have little to no effect on binding, reinforcing the modest role that direct hydrogen bond contacts assume in this interaction, when compared with nonbonded contacts. When comparing *Bc*CohScaA1_11_-DocCel48’s X-ray structure with a modeled one where the Doc was rotated 180° relative to the cohesin, it was observed that most contacts are maintained and no significant clashes were found (Fig. 3*B*) suggesting that, similarly to type I dockerins in *C. thermocellum* and *B. cellulosolvens* itself (6, 10, 22), this type II dockerin can interact with the cohesin in two distinct orientations. To test this hypothesis, the critical methionine and phenylalanine were replaced in only one of the dockerin repeats (*Bc*DocCel48 mutants M1, M2, F27A, and F60A). ITC assays performed using these variants against the cohesin revealed that binding was still possible (Table 3), underpinning the presence of the dual-binding mode, which compensates for the mutations.

**Table 3:** Thermodynamics of interaction between wild-type *Bc*CohScaA1 and various variants of *Bc*DocCel48

Dockerin	K_a_ M^−1^	ΔG° kcal mol^−1^	ΔH kcal mol^−1^	−TΔS° kcal mol^−1^	N
WT	3.31E7 ± 1.15E6	−10.63	−15.17 ± 0.27	4.54	0.98
M1 (M17S/A18D)	1.11E7 ± 7.39E6	−9.97	−16.02 ± 0.68	6.05	0.98
M2 (M50S/A51D) (Structure)	1.41E6 ± 1.49E5	−8.7	−17.73 ± 0.28	9.03	0.99
M1 + M2	Nb	Nb	Nb	Nb	Nb
M17A/M50A	Nb	Nb	Nb	Nb	Nb
N16A/N49A	1.36E5 ± 1.66E4	−7.27	−14.09 ± 1.01	6.82	0.99
M21A/I54A	1.20E6 ± 2.85E5	−8.57	−9.30 ± 0.24	0.73	1.00
Q25A/Q58A	1.35E6 ± 2.25E5	−8.64	−8.86 ± 0.39	0.22	0.99
F27A/F60A	Nb	Nb	Nb	Nb	Nb
M1 + F27A	Nb	Nb	Nb	Nb	Nb
M1 + F60A	5.84E4 ± 6.99E3	−6.89	−60.92 ± 33.91	54.03	1.01
M2 + F27A	4.45E5 ± 3.03E4	−7.98	−13.23 ± 0.31	5.25	1.01
M2 + F60A	Nb	Nb	Nb	Nb	Nb

Nb, No binding.

All thermodynamic parameters were determined at 308 K.

The structure reported here shows that, by mutating the methionine at position 50, it is possible to force the dockerin to interact through a single interface, resulting in the protein homogeneity required for crystallization. Also, ITC experiments performed with *Bc*DocCel48 mutant derivatives that had only one of the repeated methionine residues changed (mutants M1 and M2) have resulted in affinity values similar to those obtained with the wild-type dockerin. Besides supporting the dual-binding mode, this suggests that methionine residues 17 and 50 participate in binding in an alternating fashion, with either one or the other establishing important contacts with the cohesin, depending on the dockerin orientation. On the other hand, the data suggest that both Phe27 and Phe60 interact with the cohesin. ITC experiments with the F27A and F60A mutants show a drop in affinity, even though binding is still observed, suggesting that they both contribute to the two binding modes. To evaluate whether the relative importance of each phenylalanine is also dependent on dockerin orientation, four dockerin mutants were produced (M17S/F27A, M17S/F60A, M50S/F27A, and M50S/F60A) and had their cohesin affinity tested by ITC. The reasoning behind the mutant design was to use the methionine mutations to force the dockerin into a specific orientation and then test each phenylalanine independently. The results have shown that, when the dockerin interacts with its N-terminal critical methionine (Met17), changing the N-terminal phenylalanine causes a drop in affinity similar to that of the single phenylalanine mutants, while mutating the C-terminal phenylalanine (Phe60) completely abrogates binding. The inverse is true for a Met50-dominated interaction. This means that the phenylalanine on the contralateral helix to that of the binding methionine assumes a prominent role, providing a vital contribution to binding or, in other words, cohesin binding is not possible without the presence of both Met17 and Phe60 or both Met50 and Phe27 (Fig. S2). Rather than having a clear dominant helix interaction, like in classical dual-binding mode type I complexes, or two equally contributing helices as in *C. thermocellum*’s single-binding mode type II interactions, DocCel48’s binding surface seems to have a dominating “side” for each binding orientation, with major contributions from the N-terminal end of helix-1 and the C-terminal end of helix-3, or vice versa.

### The ubiquity of the dual-binding mode

The role of the dual-binding mode in the cohesin–dockerin interaction has long been a topic of discussion. The most consensual hypothesis is that it provides an extra degree of flexibility that allows the accommodation of numerous cellulosomal components in close proximity, avoiding steric hindrance by placing enzymes and structural cellulosomal elements in the correct orientation (6, 23). When it was first reported in the cellulosome of *C. thermocellum*, the dual-binding mode was thought to be exclusive to type I Coh-Doc complexes. In this species, type I interactions are responsible for assembling the enzymatic components into the main scaffoldin *via* a dual-binding mode, whereas type II interactions occur between the main scaffoldins and cell-anchoring scaffoldins through single-binding mode contacts. The same was observed in other cellulosomes, such as that of *Clostridium (Hungateiclostridium) cellulolyticum*, leading to the assumption that enzyme recruiting would require more flexibility than cell-wall anchoring (23). Later, it was revealed that in the cellulosome of *B. cellulosolvens* there is a role reversal in the Coh-Doc types, with type I complexes working in scaffoldin assembling rather than enzyme recruiting. Nonetheless, *B. cellulosolvens’*type I dockerins are also able to interact with their cognate partners in two distinct orientations (10). In this context, the meaning of “reversed” types in *B. cellulosolvens* would indicate a higher requirement in flexibility for scaffoldin assembly rather than for enzyme recruiting. The results presented here seem to suggest that the dual-binding mode exists also in type II *B. cellulosolvens* Coh-Doc complexes. This contradicts previous notions that the dual-binding mode is either type- or function-dependent, as there are both type I and II complexes with dual-binding mode as well as enzyme recruiting and cell-surface anchoring/scaffoldin assembling complexes displaying this property. In fact, excluding the somewhat obscure “Group-R” Coh and Docs whose function and binding mechanism remain unknown, it seems that *B. cellulosolvens* cellulosome is assembled exclusively through dual-binding mode interactions.

Although there is a comprehensive structural and mechanistic understanding of Coh-Doc protein–protein interactions, the biological relevance of the dual-binding mode remains elusive. One hypothesis is that the intrinsic symmetry in Docs may result from gene duplication without functional relevance, apart from extending the Coh-Doc contact surface. The fact that *R. flavefaciens* can assemble its cellulosome exclusively through single-binding mode interactions (11, 24, 25) and the identification of functional nonduplicated “half-dockerins” in this species (26) support this notion and are strong arguments against the relevance of the dual-binding mechanism. Nonetheless, *R. flavefaciens*, unlike other cellulosome secreting species, inhabits a relatively stable environment with little temperature and pH fluctuations. Furthermore, it assembles a relatively small, albeit diverse, cellulosome, meaning that steric hindrance might not impose significant selective pressure for the evolution of highly dynamic Coh-Doc interactions. Thus, the hypothesis that the dual-binding mode could persist throughout evolution without presenting selective advantage is a controversial one. Also, recent evidence suggests the presence of regulatory mechanisms that dictate dockerin-binding orientation (27, 28). Whether by the influence of external factors, such as pH, or through the action of enzymes that alter the isomerization state of key dockerin residues, cellulosomes appear to be able to control the switch between dockerin-binding platforms and even display preference for one Doc-binding platform over the other (27, 28). This active regulation of cellulosome plasticity points to a functional relevance for the dual-binding mode, although further work is still required to address the premise that the dual-binding mode ultimately serves as a decluttering mechanism. Notwithstanding, given the unprecedented complexity of *B. cellulosolvens* system, with over 200 dockerin-bearing components and 78 cohesin modules scattered across 31 scaffoldins supporting the assembly of up to 110 enzymes in a single unit, it is likely that the added flexibility in both enzyme recruiting and scaffoldin assembly would result in an evolutionary advantage.

## Conclusions

The present study revealed the structure and organization of the type II Coh-Doc complex interaction responsible for enzyme incorporation into the main scaffoldin ScaA of *B. cellulosolvens* cellulosome. The structure of this complex displays typical folds for both the cohesin and dockerin modules, similar to that found in the vast majority of Coh-Doc structures, although the binding interface presents some significant particularities. Typically, in the classic type I complexes, one Doc helix is further away from the Coh, while the other dominates the interaction. Contrastingly, in type II complexes both helices make equal contribution to the binding. The present complex seems to stand somewhere in between the two, with both helices making important contributions to cohesin recognition while there is still a clear dominant “side.” Computational modeling as well as binding studies indicates that the two modules interact *via* a dual-binding mode, thus imparting the dual-binding mode as a general feature of all type I and II interactions in *B. cellulosolvens*. This means that the assembly of *B. cellulosolvens* cellulosome relies solely on cohesin–dockerin complexes with a dual-mode of binding. Considering the incredible complexity of this system and its capacity to assemble up to 110 enzymes in a single unit, it is not surprising that this cellulosome has evolved mechanisms toward acquiring a higher degree of flexibility and plasticity that would allow the accommodation of such a large number of enzymes and structural components in close proximity. Recent evidence suggests that dockerin-binding orientation can be regulated through changes in the biochemistry of the surrounding environment or by altering the isomerization of certain proline residues. This active regulation of cellulosome plasticity suggests that, indeed, the dual-binding mode plays a fundamental role in cellulosome function. Nonetheless, the premise that avoiding steric hindrance of tightly packed cellulosomal enzymes is the ultimate goal for the dual-binding mode is one that requires further exploration.

## Experimental procedures

### Gene synthesis and DNA cloning

Docs are inherently unstable when produced in *E. coli*. To promote Doc stability, *B. cellulosolvens* Doc of protein WP_050753099 (residues 683–752) was coexpressed *in vivo* with the 11th Coh of ScaA1, *Bc*CohScaA1_11_ (AAG01230; residues 2073–2242) (29, 30). The immediate binding of *Bc*DocCel48 to *Bc*CohScaA1_11_ is believed to confer the necessary Doc stabilization. The genes encoding the two proteins were designed with a codon usage optimized to maximize expression in *E. coli*, synthesized *in vitro* (GenScript Ltd), and cloned into pET28a (Merck Millipore) under the control of separate T7 promoters. The *Bc*DocCel48-encoding gene was positioned at the 5’ end and the *Bc*CohScaA1_11_-encoding gene at the 3’ end of the artificial DNA. A T7 terminator sequence (to terminate transcription of the Doc gene) and a T7 promoter sequence (to control transcription of the Coh gene) were incorporated between the sequences of the two genes. This construct contained specifically tailored NheI and NcoI recognition sites at the 5’ end and XhoI and SalI at the 3’ end to allow subcloning of the nucleic acid into pET-28a (Merck Millipore) such that the sequence encoding a six-residue His tag could be introduced either at the N-terminus of the Doc (through digestion with NheI and SalI, incorporating the additional sequence MGSSHHHHHHSSGLVPRGSHMAS at the N-terminus of the *Bc*DocCel48) or at the C-terminus of the *Bc*CohScaA1_11_ (by cutting with NcoI and XhoI, which incorporates the additional sequence LEHHHHHH at the C-terminus of the Coh). To block the dual-binding mode and promote the structural homogeneity required for protein crystallization, two different genes were synthetized, each with a distinct Doc mutant: mutant M1 with the M17S and A18D amino acid changes and mutant M2 with the M50S and A51D replacements. These substitutions mimic the Coh recognition residues present in Type I Docs of *B. cellulosolvens*, which do not cross-bind with type II Cohs such as the ones in ScaA1 (4). Thus, as a result of this strategy, four pET28a plasmid derivatives were produced: M1 and M2 variants with the engineered tag either in the Doc or the Coh module. The four plasmids were used to express *Bc*CohScaA1_11_-DocCel48 M1 and M2 complexes in *E. coli*. Recombinant *Bc*DocCel48 and *Bc*CohScaA1_11_ primary sequences are presented in Table S2.

To produce recombinant *Bc*DocCel48 and *Bc*CohScaA1_11_ individually, the sequences encoding each of the two modules were amplified from *B. cellulosolvens* genomic DNA by PCR, using NZYProof polymerase (NZYTech Ltd) and the primers shown in Table S3. Following gel purification, the *Bc*DocCel48 encoding amplicon was inserted into the pHTP8 plasmid by homologous recombination (NZYTech Ltd). The resulting expressed product consists of His-tagged *Bc*DocCel48 fused to *Trx*A for increased solubility and stability. The *Bc*CohScaA1_11_ encoding gene was cloned into pET28a after digestion with NcoI and XhoI restriction enzymes.

To produce the mutants used in the binding experiments, several TrxA-*Bc*DocCel48 protein derivatives were produced using site-directed mutagenesis (Table S3). Each of the newly generated gene sequence was fully sequenced to verify that only the desired mutation accumulated in the nucleic acid chain.

### Protein expression and purification

As higher yields were obtained with the cohesin-tagged complexes, these were the ones used in further crystallography experiments. The complex was purified in three steps using an AKTA FPLC machine. The first step was Immobilized Metal-ion Affinity Chromatography (IMAC) purification in a HisTrap HP 5 ml column (GE Healthcare). The column was equilibrated with 50 mM NaHepes buffer, pH 7.5, containing 1 M NaCl, 10 mM imidazole, and 5 mM CaCl_2_. Proteins were eluted from the column with an imidazole gradient ranging from 10 mM to 300 mM over 25 column volumes. The fractions containing the protein–protein complexes were selected based on SDS-PAGE analysis. The IMAC-purified proteins were then buffer-exchanged in PD-10 Sephadex G25 M gel filtration columns (GE Healthcare) into 20 mM Tris-HCl buffer, pH 8.0, and 2 mM CaCl_2_. The excess unbound cohesin in the sample was removed with a subsequent purification step by anion exchange chromatography using a column loaded with Source 30Q media (GE Healthcare). The separation of the individual proteins from the complex was achieved through the application of a 0 to 1 M NaCl elution gradient. Prior to filtration chromatography, the protein fractions were buffer-exchanged into 20 mM NaHepes buffer, pH 7.5, containing 200 mM NaCl and 2 mM CaCl_2_. The purity of the protein was confirmed by SDS-PAGE analysis of the collected fractions. The purified protein was concentrated with Amicon centricons with 10-kDa molecular-mass cutoff centrifugal membranes (Millipore) by centrifuging at 5000 rpm at 4 °C. The final concentration of the protein was kept around 25 mg/ml. A final purification step was performed through size-exclusion chromatography using a HiLoad 16/60 Superdex 75 column (GE Healthcare) previously equilibrated with 20 mM NaHepes buffer, pH 7.5, containing 200 mM NaCl and 2 mM CaCl_2_. Pure complexes were buffer-exchanged by washing with 2 mM CaCl_2_ and concentrated to 50 mg/ml.

### Complex crystallization

The Type II complex Coh-Doc of *B. cellulosolvens* was crystallized at 293K by the hanging drop vapor diffusion method and obtained by mixing an equal volume (1 μl) of protein (50 mg/ml in water) and reservoir solution (30% (m/v) polyethylene glycol (PEG) 4000, 0.1 M Tris-HCl, pH 8.5, and 0.2 M magnesium chloride). The crystals were grown over a period of 5 to 6 days at room temperature. Single crystals were harvested in a solution containing 35% (m/v) PEG 4000 and 0.2 M magnesium chloride, and flash-frozen in a liquid nitrogen stream at 100K, using 30% (vol/vol) of glycerol as a cryoprotectant added to the harvesting solution. Only the complex involving the M2 mutant (C-terminal) of *Bc*DocCel48 produced good-quality crystals.

### X-ray diffraction, 3D structure determination and refinement

The data were collected at 100 K, using 0.934 Å wavelength radiation at the European Synchrotron Radiation Facility (ESRF), ID14-EH1 to a maximum resolution of 1.90 Å. Diffraction data were processed and scaled, respectively, with programs MOSFLM (31) and SCALA (32) from the CCP4 suite (33). The Matthews coefficient of the ScaA type II Coh-Doc crystal is 1.91 Å^3^ Da^−1^ for two 27.5 kDa heterodimers in the asymmetric unit, with a solvent content of 35.7%. The space group was determined to be *P*12_1_1 with unit cell dimensions: *a* = 43.4 Å, *b* = 116.1 Å, *c* = 45.2 Å, with β = 112.45° (Table 1).

Considering the calculated Matthews coefficient and because there was no dockerin structure available from the cellulosome of *B. cellulosolvens*, molecular replacement attempts were performed searching just for two copies of the cohesin module in the monoclinic *P*2 cell. The previously described and available crystal structure of the ScaA type II cohesin module from *B. cellulosolvens*, with accession code 1tyj (15), was used as search model for molecular replacement. The Patterson search was done with program PHASER (34), implemented in the CCP4 interface (33), and a clear solution with two cohesins in the asymmetric unit was found in space group *P*2_1_.

Initial building of the structures into the electron density as well as building of the dockerin modules was performed using the software ARP/wARP (19) and any remaining residues were built interactively using program COOT (20). Model refinement and electron density map calculations were done with program REFMAC5 (21) from the CCP4 suite (33). The final round of refinement was performed using the TLS/restrained refinement procedure using each module as a single group. The root mean square deviation of bond lengths, bond angles, torsion angles, and other indicators were continuously monitored using validation tools in COOT and MOLPROBITY (35). The final model has R_work_ = 16.3% and R_free_ = 22.5% and includes 299 water molecules and four calcium ions. Due to disorder, residues Met1, Ala2, and the 6 C-terminal histidine residues of chain A (cohesin), stretches Gly32-Asn37 and Ala66-Phe71 of chain B (dockerin), Met1, Ala2, Leu174, Glu175, and the 6 C-terminal histidine residues of chain C (cohesin) and Ala30-Asn44 and Ser65-Phe71 of chain D (dockerin) could not be built. The structure is available in the Protein Data Bank under the accession code 2y3n.

### Structural alignments

Structural alignments were performed with the Matchmaker tool of UCSF Chimera using the Needleman-Wunsch algorithm and BLOSUM62 matrix (36).

### Isothermal titration calorimetry (ITC)

All ITC experiments were carried out at 308 K. The purified wild-type *Bc*DocCel48 and mutant variants and *Bc*CohScaA1_11_ were diluted to the required concentrations and filtered using a 0.45-μm syringe filter (PALL). During titrations, the Doc constructs were stirred at 307 revolutions/min in the reaction cell and titrated with 28 successive 10 μl injections of *Bc*CohScaA1_11_ at 220-s intervals. Integrated heat effects, after correction for heats of dilution, were analyzed by nonlinear regression using a single-site model (Microcal ORIGIN version 7.0, Microcal Software). The fitted data yielded the association constant (K_A_) and the enthalpy of binding (Δ*H*). Other thermodynamic parameters were calculated using the standard thermodynamic equation: ΔRTln*K*_A_ = Δ*G* = Δ*H* − TΔ*S*.

### Nondenaturing gel electrophoresis

For the NGE experiments each of the *Bc*DocCel48 and *Bc*DocCel48 variants, at a concentration of 30 μM, was incubated in the presence and absence of 30 μM *Bc*CohScaA1_11_ for 30 min at room temperature and separated on a 10% native polyacrylamide gel. Electrophoresis was carried out at room temperature. The gels were stained with Coomassie Blue. Complex formation was detected by the presence of an additional band displaying a distinct electrophoretic mobility from the one presented by the individual modules.

## Data availability

Coordinates and structure factors have been deposited in the Protein Data Bank under accession code PDB 2Y3N [https://www.rcsb.org/structure/2Y3N]. All further data supporting the findings of this study are available from the corresponding author, upon reasonable request.

## Supporting information

This article contains supporting information.

## Conflict of interest

The authors declare that they have no conflicts of interest with the contents of this article.
