# Supplementary material for: A dual cohesin–dockerin complex binding mode in Bacteroides cellulosolvens contributes to the size and complexity of its cellulosome
Source: J Biol Chem. 2021 Mar 18;296:100552. doi: 10.1016/j.jbc.2021.100552 (PMC8063739; doi:10.1016/j.jbc.2021.100552)
Supplement: Supplemental Figures S1–S2 and Tables S1–S3 [file mmc1.pdf]

A dual cohesin–dockerin complex binding mode in *Bacteroides cellulosolvens* contributes to the size and complexity of its cellulosome

Marlene Duarte[a, *], Aldino Viegas[b, *], Victor D. Alves[a], José A.M. Prates[a], Luís M.A. Ferreira[a], Shabir Najmudin[c], Eurico J. Cabrita[b], Ana Luísa Carvalho[b, †], Carlos M.G.A. Fontes[a,d] and Pedro Bule[a, †]

[a] *CIISA – Centre for Interdisciplinary Research in Animal Health, Faculty of Veterinary Medicine, University of Lisbon, Pólo Universitário do Alto da Ajuda, Avenida da Universidade Técnica, 1300-477 Lisboa, Portugal;*

[b] *UCIBIO, Departamento de Química, Faculdade de Ciências e Tecnologia, Universidade Nova de Lisboa, 2829-516 Caparica, Portugal;*

[c] *Randall Centre for Cell and Molecular Biophysics, King's College London, 3rd Floor, New Hunt's House, Guy's Campus, London SE1 1UL, United Kingdom;*

[d] *NZYTech genes & enzymes, Estrada do Paço do Lumiar, 1649-038 Lisboa, Portugal.*

[*] These authors have made equal contributions to this work

[†] Correspondence and requests for materials should be addressed to PB (email: pedrobule@fmv.ulisboa.pt) or to ALC (email: almc@fct.unl.pt)

Running title: Type II *Bacteroides cellulosolvens* Coh-Doc complex

Keywords: Cellulosome, cohesin, dockerin, dual-binding, protein complex, crystal structure, cellulose, cellulase

List of Contents

Supporting Table 1

Supporting Table 2

Supporting Table 3

Supporting Figure 1

Supporting Figure 2

SUPPORTING TABLES

Table S1. Main hydrophobic contacts between *Bc*CohScaA1$_{11}$ and *Bc*DocCel48. Table was made using data retrieved from the PDBSUM server. Some of the dockerin residues are marked as belonging either to helix 1 (H1) or to helix 3 (H3) interfaces.

Dockerin					CohScaA1$_{11}$
		**Residue**	**Residue #**		**Residues**
		Asp	12	< >	Asn141 (6),
		Val	14	< >	Ser139 (2), Asn141 (2)
H1		Asn	16	< >	Gln37 (3), Ser139 (3), Met148 (2)
H1		Met	17	< >	Tyr36, Gln37 (5), Gly92 (2), Arg93, Phe150 (2)
H1		Ala	18	< >	Phe150
H1		Val	20	< >	Leu94 (2), Met96
H1		Met	21	< >	Ser34, Gly35, Leu94 (3), Phe150 (8), Gly154
H1		Leu	23	< >	Met96
H1		Ala	24	< >	Leu94, Met96 (7), Leu98 (3)
H1		Gln	25	< >	Leu98, Asp153 (4), Gly154 (3)
		Phe	27	< >	Asn75 (3), Met96 (10)
	H3	Ala	53	< >	Met79
	H3	Ala	57	< >	Met79 (3)
	H3	Phe	60	< >	Gln37 (4), Met79 (6), Lys81 (5), Asn90 (4)
	H3	Gly	61		Lys81 (3), Ser139 (4)

Table S2. Recombinant protein sequences of *BcCohScaBA*$_{11}$, *BcDocCel48* and respective mutant variants produced for the crystallization and interaction studies. The mutated residues are highlighted in black.

Protein	Vector	Sequence
*Bc*CohScaA1$_{11}$-DocCel48 M2 (structure)	pET28a	MASGSVLTAIDNDKVAVGDKVTLTINVDKITNFSGYQFNIKYNTTYLQPWDTIADEAYTDSTMPDYGTLLQGRFNATDM<br>SKHNLSQGVLNFGRLYMNLSAYRASGKPESTGAVAKVTFKVIKEIPAEGIKLATFENGSSMNNAVDGTMLFDWDGNMYS<br>SSAYKVVQPGLIYPKLEHHHHHH<br><br>MFVKLKGDLNGDGVINMADVMILAQSFGKAIGNPGVNEKADLNNDGVIN[redacted]DAIILAQYFGKTKSAEVVMF
TrxA-*Bc*DocCel48 WT	pHTP8	MGSDKIIHLTDDSFDTDVLKADGAILVDFWAEWCGPCKMIAPILDEIADEYQGKLTVAKLNIDQNPGTAPKYGIRGIPT<br>LLLFKNGEVAATKVGALSKGQLKEFLDANLAGSAMGSSHHHHHHSSGPQQGLRFVKLKGDLNGDGVINMADVMILAQSF<br>GKAIGNPGVNEKADLNNDGVINMADAIILAQYFGKTKSAEVVMF
TrxA-*Bc*DocCel48 M1 (M17S, A18D)	pHTP8	...FVKLKGDLNGDGVIN[redacted]DVMILAQSFGKAIGNPGVNEKADLNNDGVINMADAIILAQYFGKTKSAEVVMF
TrxA-*Bc*DocCel48 M2 (M50S, A51D)	pHTP8	...FVKLKGDLNGDGVINMADVMILAQSFGKAIGNPGVNEKADLNNDGVIN[redacted]DAIILAQYFGKTKSAEVVMF
TrxA-*Bc*DocCel48 M1 + M2	pHTP8	...FVKLKGDLNGDGVIN[redacted]DVMILAQSFGKAIGNPGVNEKADLNNDGVIN[redacted]DAIILAQYFGKTKSAEVVMF
TrxA-*Bc*DocCel48 N16A / N49A	pHTP8	...FVKLKGDLNGDGVI[redacted]MADVMILAQSFGKAIGNPGVNEKADLNNDGVI[redacted]MADAIILAQYFGKTKSAEVVMF
TrxA-*Bc*DocCel48 M17A / M50A	pHTP8	...FVKLKGDLNGDGVIN[redacted]ADVMILAQSFGKAIGNPGVNEKADLNNDGVIN[redacted]ADAIILAQYFGKTKSAEVVMF
TrxA-*Bc*DocCel48 M21A / I54A	pHTP8	...FVKLKGDLNGDGVINMADV[redacted]ILAQSFGKAIGNPGVNEKADLNNDGVINMADA[redacted]ILAQYFGKTKSAEVVMF
TrxA-*Bc*DocCel48 Q25A / Q58A	pHTP8	...FVKLKGDLNGDGVINMADVMILA[redacted]SFGKAIGNPGVNEKADLNNDGVINMADAIILA[redacted]YFGKTKSAEVVMF
TrxA-*Bc*DocCel48 F27A / F60A	pHTP8	...FVKLKGDLNGDGVINMADVMILAQS[redacted]GKAIGNPGVNEKADLNNDGVINMADAIILAQY[redacted]GKTKSAEVVMF
TrxA-*Bc*DocCel48 M1 + F27A	pHTP8	...FVKLKGDLNGDGVIN[redacted]DVMILAQS[redacted]GKAIGNPGVNEKADLNNDGVINMADAIILAQYFGKTKSAEVVMF
TrxA-*Bc*DocCel48 M1 + F60A	pHTP8	...FVKLKGDLNGDGVIN[redacted]DVMILAQSFGKAIGNPGVNEKADLNNDGVINMADAIILAQY[redacted]GKTKSAEVVMF
TrxA-*Bc*DocCel48 M2 + F27A	pHTP8	...FVKLKGDLNGDGVINMADVMILAQS[redacted]GKAIGNPGVNEKADLNNDGVIN[redacted]DAIILAQYFGKTKSAEVVMF
TrxA-*Bc*DocCel48 M2 + F60A	pHTP8	...FVKLKGDLNGDGVINMADVMILAQSFGKAIGNPGVNEKADLNNDGVIN[redacted]DAIILAQY[redacted]GKTKSAEVVMF
*Bc*CohScaA1$_{11}$ WT	pET28a	MASGSVLTAIDNDKVAVGDKVTLTINVDKITNFSGYQFNIKYNTTYLQPWDTIADEAYTDSTMPDYGTLLQGRFNATDM<br>SKHNLSQGVLNFGRLYMNLSAYRASGKPESTGAVAKVTFKVIKEIPAEGIKLATFENGSSMNNAVDGTMLFDWDGNMYS<br>SSAYKVVQPGLIYPKLEHHHHHH

Table S3. Set of primers used to isolate the *Bc*DocCel48 M2 gene, to revert *Bc*DocCel48 M2 to the WT and to generate the remaining mutant derivatives. Mutated codons are shown bold and underlined.

ID	Primers used
***Bc*DocCel48 M2 isolation**	5′ tcagcaagggctgaggtttgtgaaactgaaaggc<br>3′ tcagcggaagctgaggttagaacataacgacttccgc
***Bc*DocCel48 WT**	5′ gacggcgttatcaac**atggcc**gacgctatcatcctg<br>3′ caggatgatagcgtc**ggccat**gttgataacgccgtc
***Bc*DocCel48 M1**	5′ gatggtgttatcaac**tcggac**gatgttatgatcctg<br>3′ caggatcataacatc**gtccga**gttgataacaccatc
***Bc*DocCel48 N16A**	5′ ggcgatggtgttatc**gcc**atggccgatgttatg<br>3′ cataacatcggccat**ggc**gataacaccatcgcc
***Bc*DocCel48 M17A**	5′ gatggtgttatcaac**gcg**gccgatgttatgatc<br>3′ gatcataacatcggc**cgc**gttgataacaccatc
***Bc*DocCel48 M21A**	5′ caacatggccgatgtt**gcg**atcctggctcaatc<br>3′ gattgagccaggat**cgc**aacatcggccatgttg
***Bc*DocCel48 Q25A**	5′ gttatgatcctggct**gca**tcttttggtaaagc<br>3′ gctttaccaaaaga**tgc**agccaggatcataac
***Bc*DocCel48 F27A**	5′ gatcctggctcaatct**gct**ggtaaagcaattggc<br>3′ gccaattgctttacc**agc**agattgagccaggatc
***Bc*DocCel48 N49A**	5′ caatgacggcgttatc**gcc**atggccgacgctatc<br>3′ gatagcgtcggccat**ggc**gataacgccgtcattg
***Bc*DocCel48 M50A**	5′ gacggcgttatcaac**gcg**gccgacgctatcatc<br>3′ gatgatagcgtcggc**cgc**gttgataacgccgtc
***Bc*DocCel48 I54A**	5′ caacatggccgacgct**gcc**atcctggctcagtatttc<br>3′ gaaatactgagccaggat**ggc**agcgtcggccatgttg
***Bc*DocCel48 Q58A**	5′ gctatcatcctggct**gcg**tatttcggtaaaacc<br>3′ ggttttaccgaaata**cgc**agccaggatgatagc
***Bc*DocCel48 F60A**	5′ catcctggctcagtat**gcc**ggtaaaaccaaaagtg<br>3′ cacttttggttttacc**ggc**atactgagccaggatg

SUPPORTING FIGURES

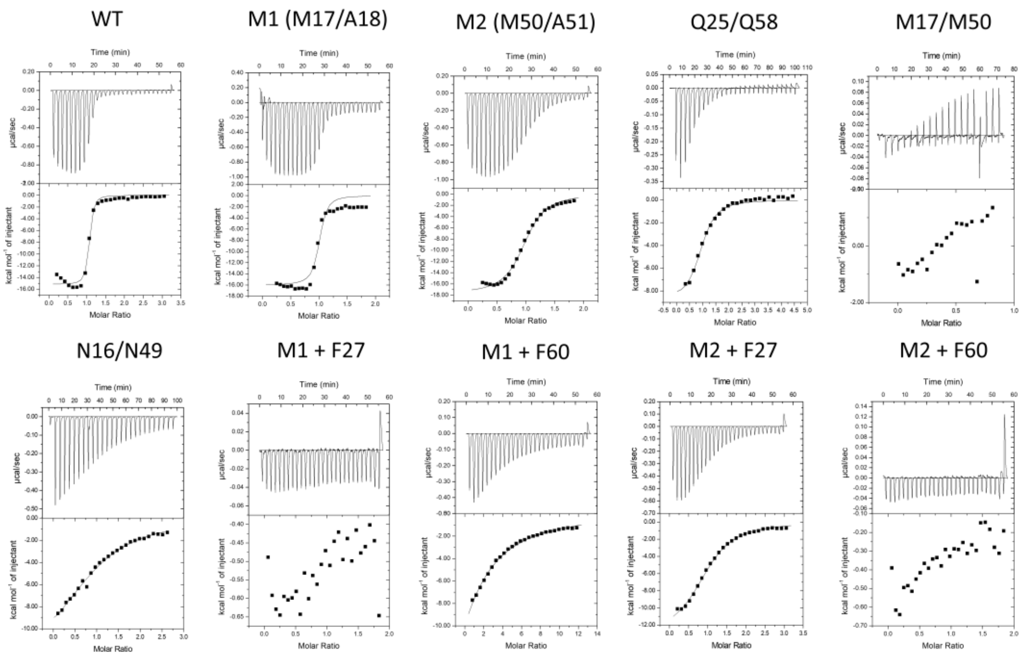

Figure S1. Binding affinity of *Bc*CohScaA1$_{11}$ to *Bc*DocCel48 wild type and mutant derivatives as determined by ITC. Example binding isotherms for *Bc*CohScaA1$_{11}$ vs *Bc*DocCel48 wild type and mutants, for determination of key interacting residues and evaluation of the dual binding mode. The upper part of each panel shows the raw heats of binding, whereas the lower parts comprise the integrated heats after correction for heat of dilution. The curve represents the best fit to a single-site binding model. The corresponding thermodynamic parameters are shown in Table 3.

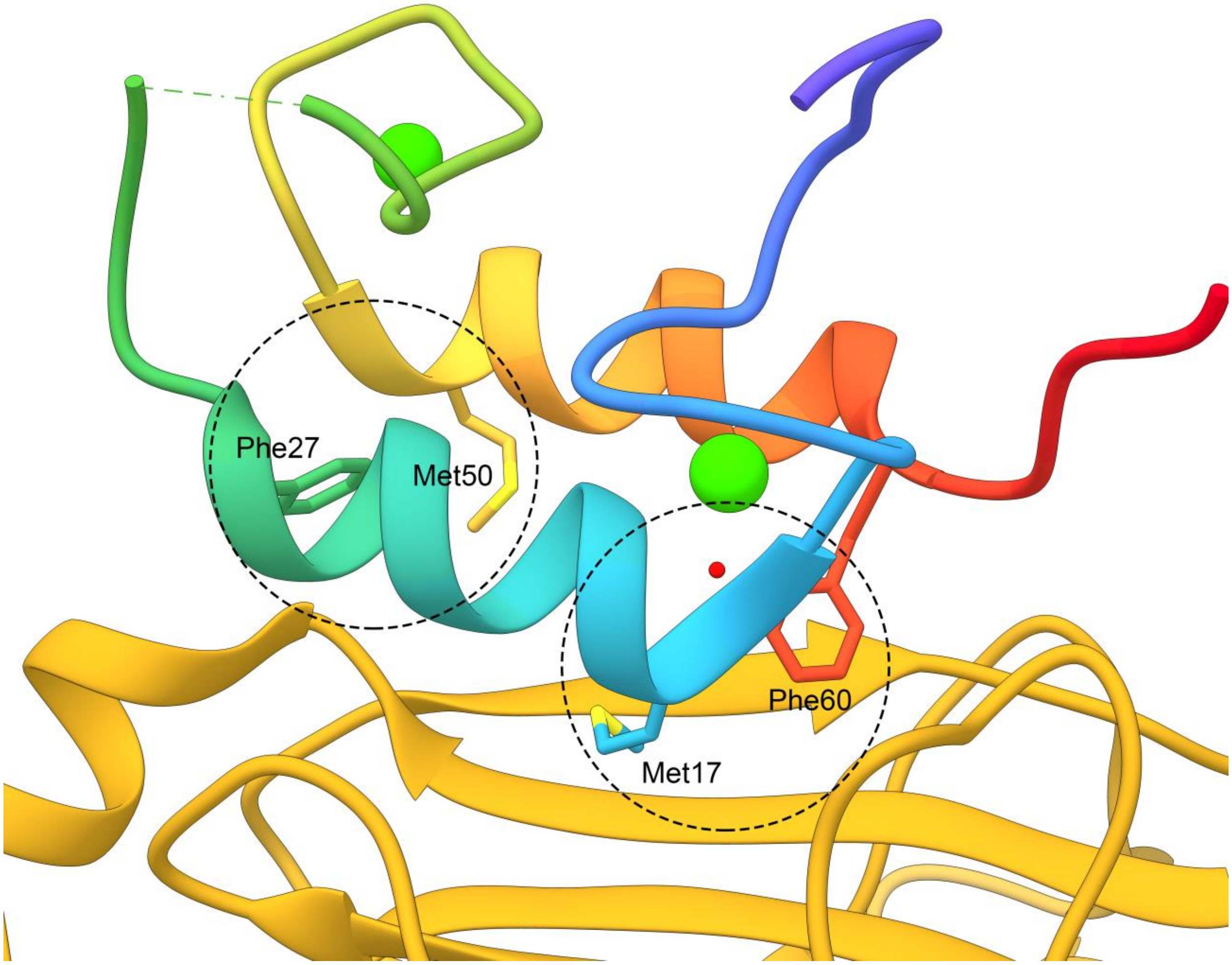

Figure S2. Critical dockerin residues for cohesin recognition. As shown by the ITC data, binding of *Bc*DocCel48 and *Bc*CohScaA1$_{11}$ only occurs in the presence of either the Met17/Phe60 pair or Met50/Phe27 pair (or both). Removal of one or both members of one pair still allows binding through the opposite interface, while removing one member from each pair is enough to abrogate binding. The dockerin is shown in ribbon representation, color-ramped from *N-terminus* (blue) to *C-terminus* (red) and the cohesin is colored in gold. The key residue pairs are shown in stick representation and highlighted with a dashed black circle.
